# Transferable and Transparent Energy Decomposition-Based Machine Learning Models for Computing Accurate Reaction Energetics

**DOI:** 10.1021/acs.jctc.5c01184

**Published:** 2025-10-29

**Authors:** Carlos R. Jacinto-Mejía, Loriano Storchi, Giovanni Bistoni

**Affiliations:** † Department of Chemistry, Biology and Biotechnology, University of Perugia, Perugia 06123, Italy; ‡ Dipartimento di Farmacia, Universita’ G. D’Annunzio, Chieti-Pescara 66100, Italy

## Abstract

We present a transferable, interpretable, and modular machine-learning framework that enhances the accuracy of density functional theory (DFT) reaction energies using physically meaningful energy-decomposition descriptors. Reaction energies computed at the DFT level with standard basis sets are first decomposed into chemically intuitive contributions—such as kinetic and potential energy—which are then used to train a library of linear regression (LR) models. This includes a general-purpose model that reduces mean absolute percentage errors (MAPE) relative to gold standard CCSD­(T)/CBS reference values by up to 63% compared to uncorrected DFT across extended benchmark sets. In parallel, a series of specialized LR models provide improved accuracy for specific reaction classes. A random forest (RF) classifier dynamically selects the optimal model for each case, pushing accuracy further and achieving a MAPE reduction of up to 123 percentage points, all while maintaining full model interpretability. In a rigorous out-of-distribution stress test on the WCCR10 data set—containing transition-metal complexes absent from training—both the general LR model and the RF/LR pipeline retain robust performance. Unlike typical neural network models, which often face generalization challenges beyond their training set, our framework maintains stable performance outside its training domain.

## Graphical abstract

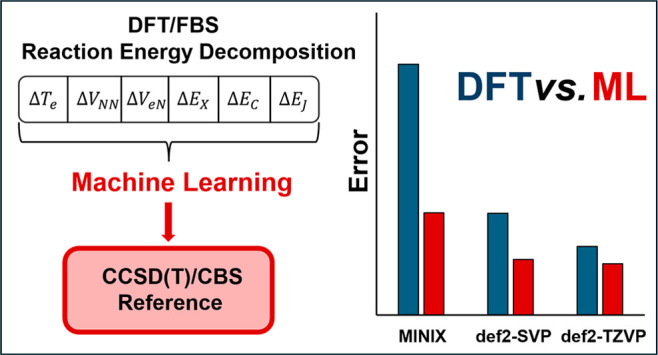

## 1 Introduction

Machine Learning (ML) has revolutionized the natural sciences, and chemistry has been at the forefront of such revolution.^1^ In particular, the integration of ML into computational chemistry has opened new directions for addressing long-standing challenges in accuracy, efficiency, and transferability of quantum chemical predictions.^2^

For example, a long-standing obstacle in the field of computational chemistry has been the high computational cost associated with accurate quantum chemical methods, such as the “gold standard” coupled-cluster theory with single, double, and perturbative triple excitations [CCSD­(T)], which restricts their routine application to small and medium-sized systems. On the other hand, widely used methods like density functional theory (DFT), while generally more affordable, suffer from systematic errors arising from approximate exchange−correlation functionals and basis set incompleteness. These inherent limitations reduce the predictive power of computational chemistry overall.

In this context, ML has emerged over the past two decades as a promising strategy to overcome these bottlenecks. By learning corrections or surrogate models from data, ML methods have shown the potential to improve the trade-off between accuracy and computational cost, enabling near-high-level accuracy at the computational cost of more economical methods.^3,4^

One of the most successful strategies in this direction is the Δ-ML approach,^5^ where an ML model is trained to predict the difference between a low-level DFT result and a higher-level reference, such as CCSD­(T). This correction is added a posteriori to the DFT results, yielding significantly improved accuracy without additional quantum mechanical calculations. Since its introduction by von Lilienfeld and co-workers,^5^ the Δ-ML scheme has been widely generalized and applied to a range of properties, including thermochemistry, noncovalent interactions, and reaction barriers.^6–9^

Notably, the idea to combine ML with DFT predates Δ-ML. In the early 2000s, Chen and co-workers pioneered the use of artificial neural networks (ANNs) to improve DFT-derived thermochemical quantities such as heats of formation and Gibbs free energies.^10,11^ Their “Statistical Correction Approach” employed ANNs trained on molecular geometries and energy-related descriptors computed using the B3LYP functional to correct systematic errors in DFT predictions of molecular properties.^12–14^ Around the same time, Xu and co-workers proposed the “X1” scheme, which used ANN-based corrections for both heats of formation and bond energies.^15,16^ Later, Lomakina and Balabin demonstrated that ANNs trained on molecular descriptors and lower-level quantum calculations could accurately estimate high-level DFT energies.^17^

Despite this growing body of work and the proven potential of ML-based corrections, such approaches have yet to become mainstream in the computational chemistry workflow. The overwhelming majority of quantum chemical simulations reported in contemporary literature to address specific chemical problems still do not incorporate ML models for generating or refining data. This limited adoption stems from several conceptual and practical challenges. Chief among these is the trade-off between model complexity and interpretability. While complex architectures such as ANNs are considered universal estimators capable of achieving high predictive accuracy,^18^ their multilayered structure obscures the relationship between input features and predicted outputs, making it difficult to understand how predictions are formed. This is especially relevant in unexplored regions of chemical space, where training data are sparse, prompting a fundamental question: how much can we trust the output of an ANN model when applied to systems lying outside its training domain (Figure 1a)? Although statistical learning theory and error analysis provide quantitative metrics to evaluate predictive uncertainty,^19^ they do not fully eliminate this epistemic opacity.

**Figure 1:**
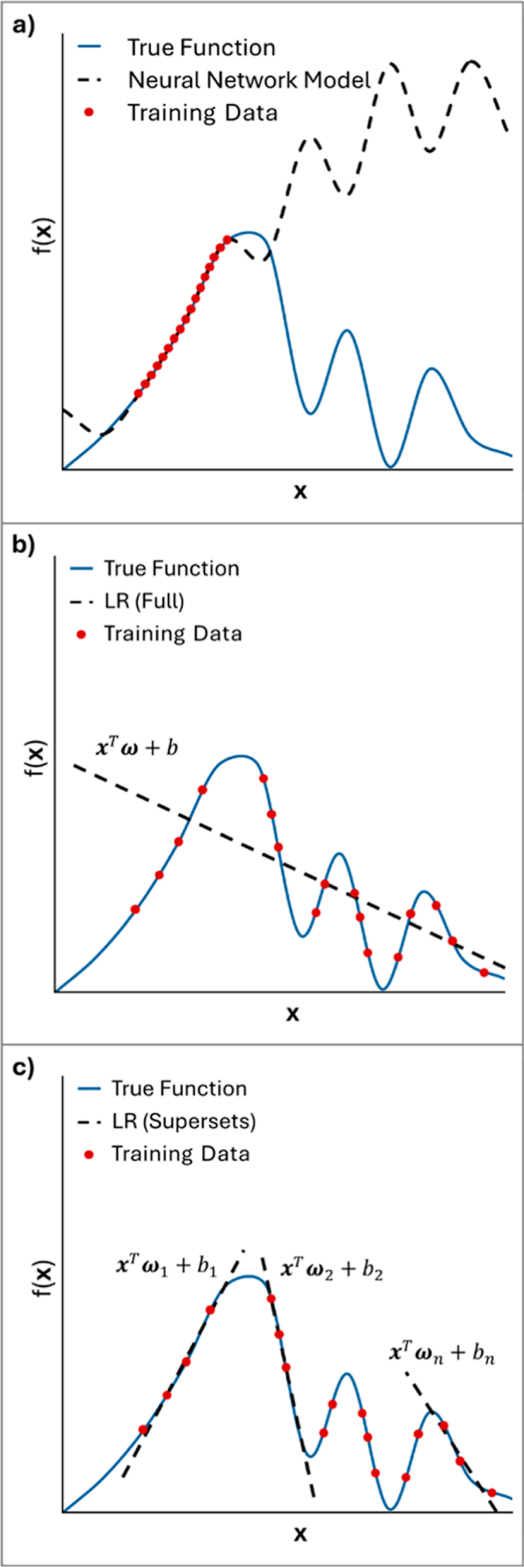
Panel (a) illustrates how ANNs, while effective interpolators, struggle with extrapolation due to their complex, nonlinear mappings. Panel (b) shows a single linear model underfitting the data, highlighting limitations of LR in capturing complex patterns. Panel (c) depicts our approach: partitioning chemical space into domains fitted by local linear models, combining interpretability with domain-specific accuracy.

In contrast, simpler models maintain a structural form that supports physical interpretability. While many sophisticated interpretable ML techniques are now available,^20^ linear regression (LR) remains a valuable option: LR models provide a transparent mapping between input features and target properties, allowing researchers to diagnose systematic failures.^21^ As a result, they remain especially valuable when chemical understanding, model transparency, and reliability are paramount. However, the price of simplicity is reduced flexibility: linear models often lack the capacity to capture subtle, nonlinear relationships in the data, which limits their overall predictive accuracy (Figure 1b).

Therefore, a central challenge in the development of ML-driven quantum chemistry remains the construction of models that strike a balance between interpretability, generalizability, and precision. While strategies like the Symbolic Regression^22^ can be adopted, a central conceptual insight that stimulated the present work is that any sufficiently smooth function can be approximated locally by a linear model—a principle that underlies classical series expansions such as the Taylor series (Figure 1c). This idea motivates our strategy: rather than attempting to learn a global, highly nonlinear mapping between features and target energies, we construct a library of simple, interpretable LR models, each trained in a specific class of reactions or within a chemically meaningful subdomain. A random forest (RF) classifier is then employed to select, for each new reaction, the most appropriate model from the library. In this way, we effectively piece together a set of local linear approximations into a flexible yet stable global framework. Unlike complex nonlinear models such as ANNs, this architecture preserves interpretability and robustness while capturing subtle domain-dependent variations in chemical behavior. Our strategy comprises three key steps:

I. Energy decomposition*.* Physically meaningful energy contributions (e.g., kinetic energy, dispersion, exchange, ...) are extracted from low-level DFT calculations on reactions drawn from extensive benchmark sets, with no computational overhead.
II. LR Models library*.* Using these extracted energy contributions as features, we train a suite of LR models on the full data set as well as on distinct subsets tailored to different reaction classes. These LR models provide transparent and chemically interpretable corrections that improve predictions toward CCSD­(T)/CBS accuracy.
III. RF classification. A RF classifier is trained on the same energy descriptors to recognize patterns in the DFT-computed features and assign each reaction to the most appropriate LR model in our library.

This RF/LR pipeline accomplishes a series of objectives simultaneously. First, it overcomes the extrapolation faltering of ANNs (Figure 1a) and gives the model better flexibility compared to a single LR prone to underfitting the data (Figure 1b). Furthermore, it dynamically partitions chemical space into regions where specific linear corrections are most effective, thus preserving interpretability and mitigating overfitting (Figure 1c).

Our numerical results show that this energy-decomposition-based hybrid RF/LR protocol improves upon DFT accuracy with no computational overhead, while also offering explicit insight into which energy components drive the corrections across different chemical regimes. Importantly, the scheme is applicable to virtually any combination of exchange–correlation functional and basis set. However, as a proof of concept, we present here only the results obtained using the PBE^23^ and PBE0 ^24^ functionals in conjunction with four basis sets of increasing quality: MINIX, which is a small basis set that covers the whole periodic table,^25^ and the def2 sets, namely def2-SVP, def2-TZVP, def2-QZVP.^26^ Thus, our model aims to correct both the intrinsic deficiencies of the exchange–correlation functional and the basis set incompleteness error (BSIE).

## 2 Results and Discussion

### 2.1 Linear Regression Models

#### 2.1.1 Technical Aspects

LR models rely on a loss function to quantify the deviation between predicted and actual values; the most common choice is the sum of squared errors (SSE), corresponding to ordinary least-squares (OLS) regression.^27^ To increase flexibility, we developed a general LR implementation that allows the use of alternative loss functions, such as the mean absolute error (MAE) and the mean absolute percentage error (MAPE). The model directly optimizes the chosen loss function using SciPy’s optimization tools,^28^ bypassing the limitations of standard OLS algorithms. It solves for the optimal coefficients in the LR expression *Y* = *X* ^*T*^ *w* + *b*, where *Y* is the target property, *X* is the matrix of descriptors, *w* is the column vector with the weights, and *b* is the intercept. The user can specify initial values for *w* and *b* to guide the optimization and select among different solvers such as BFGS or Nelder–Mead to enhance convergence robustness. In addition, L2 regularization (Ridge Regression) has been implemented in our code. This technique mitigates overfitting by adding a penalty term to the loss function, proportional to the sum of the squared coefficients. By discouraging large coefficient values, L2 regularization promotes simpler models that are less sensitive to noise in the training data and therefore generalize better to unseen data. The code is available at https://github.com/lstorchi/CLossLr.^29^

#### 2.1.2 Descriptors

Regarding the descriptors of the model, the reaction energy was decomposed in the sum of physically meaningful terms, such as kinetic energy and Coulomb repulsion, each multiplied by a weight (*w*_*i*_). Training on reaction energies rather than absolute energies not only exploits favorable error cancellation and a constrained dynamic range but also ensures that the model is selectively sensitive to the energy differences that matter most chemically. For example, core-correlation and other “spectator” contributions often remain nearly constant between reactants and products. Consequently, our model naturally emphasizes valence-region phenomena (bond breaking/formation, polarization changes, dispersion shifts) that determine reactivity and selectivity, rather than expending capacity on improving inert energy contributions.

Multiple choices are possible for an energy decomposition scheme.^30^ In the present case, the following terms were selected as energy contributions in the decomposition (the PBE-D3­(BJ) functional is used in this discussion as an illustrative example): kinetic energy *T*_*e*_ (eq 1), nuclear repulsion *V*_*NN*_ (eq 2), electron–nuclei interaction *V*_*eN*_ (eq 3), exchange energy *E*_*X*_ (eq 4), correlation energy *E*_*C*_ (eq 5), electronic Coulomb interaction *E*_*J*_ (eq 6), and the dispersion correction *E*_disp_.

1 $$T_{e}=-\frac{1}{2}\sum_{i}\int \psi_{i}^{*}\left(r\right)\nabla^{2}\psi_{i}\left(r\right)dr$$

2 $$V_{NN}=\sum_{A<B}\frac{Z_{A}Z_{B}}{\left|R_{A}-R_{B}\right|}$$

3 $$V_{eN}=-\sum_{A}Z_{A}\int \frac{\rho \left(r\right)}{\left|r-R_{A}\right|}dr$$

4 $$E_{X}^{PBE}=\int \rho \left(r\right)\epsilon_{X}^{LDA}\left(\rho \right)F_{X}\left(s\right)dr$$

5 $$E_{C}^{PBE}=\int \rho \left(r\right)\left[\epsilon_{C}^{LDA}\left(\rho ,\zeta \right)+H\left(\rho ,t,\zeta \right)\right]dr$$

6 $$E_{J}=\frac{1}{2}\iint \frac{\rho \left(r\right)\rho \left(r^{\prime }\right)}{|r-r^{\prime }|}drdr^{\prime }$$

where ρ is the electron density; ψ_*i*_(*r*) are the Kohn–Sham orbitals; *r* are position vectors for electrons in 3*D* space; *A*,*B* are indexes over nuclei; *Z*_*A*_,*Z*_*B*_ are the atomic numbers of nuclei *A*,*B*, respectively; *R*_*A*_,*R*_*B*_ are nuclear positions; ϵ _*x*_^LDA^(ρ) is the exchange energy per particle in the Local Density Approximation (LDA); *F*_*X*_(*s*) is an enhancement factor that depends on the reduced density gradient *s*; ϵ _C_^LDA^ is the correlation energy per particle in LDA that depends on ρ and on the spin polarization ζ; H is a gradient correction to the correlation that depends on the reduced gradient, *s*.^23^ For hybrid functionals, the exchange incorporates a fraction of HF exchange (eq 7). For example, PBE0 includes 75% of PBE exchange (eq 4) and 25% of HF exchange (eq 8).^24^

7 $$E_{X}^{PBE0}=\frac{1}{4}E_{X}^{HF}+\frac{3}{4}E_{X}^{PBE}$$

8 $$E_{X}^{HF}=-\frac{1}{2}\sum_{i,j}\iint \frac{\psi_{i}^{*}\left(r\right)\psi_{j}^{*}\left(r^{\prime }\right)\psi_{j}\left(r\right)\psi_{i}\left(r^{\prime }\right)}{|r-r^{\prime }|}drdr^{\prime }$$

In the training procedure, the weights were applied for all terms except for the dispersion correction (eq 9).

9 $$\Delta E_{LR}=w_{1}\Delta T_{e}+w_{2}\Delta V_{NN}+w_{3}{\Delta V}_{eN}+w_{4}\Delta E_{X}+w_{5}\Delta E_{J}+w_{6}\Delta E_{C}+{\Delta V}_{disp}+b$$

This weighted sum, calculated at a DFT-level using combinations of functionals and basis sets, was used to approximate the exact relative energy at the reference level of theory (generally, CCSD­(T)/CBS).

#### 2.1.3 Linear Regression Models Library

As a training set, we selected the GMTKN55 benchmark set,^31^ which includes 55 individual data sets covering thermochemistry, kinetics, and noncovalent interactions. This corresponds to a total of 1505 relative energies derived from 2462 single-point calculations. To extend the chemical space and explicitly account for systems involving large molecules stabilized by noncovalent interactions and/or dative bonds with a solid CCSD­(T)/CBS reference, we supplemented the training set with the LP14 benchmark set,^32^ which contains association energies of a series of medium-to-large frustrated Lewis pairs (FLPs) and classical Lewis adducts computed with a state-of-the-art computational protocol.

Using this data set, we trained LR models to correct DFT-level reaction energies by exploiting energy decomposition descriptors. We followed two strategies.

1. Global model “LR­(FULL)”: A single LR model trained on all reactions.
2. Superset-specific model “LR­(SUPERSET)”: A family of LR models, each tailored to one of the five GMTKN55 supersets—SMALL (basic thermochemistry and reaction energies of small molecules), LARGE (energetics and isomerizations of larger, more flexible systems including LP14), BARRIER (reaction barrier heights), INTER (intermolecular noncovalent interactions), and INTRA (intramolecular noncovalent interactions). Whenever we refer generically to these specialized models, we denote them as LR­(SUPERSET), with “SUPERSET” replaced by the appropriate category label. For example, the model trained on SMALL reactions is called LR­(SMALL), while that for barrier heights is LR­(BARRIER), and so on. By comparing LR­(FULL) with the suite of LR­(SUPERSET) models, we can quantify the benefit of chemical specialization and investigate how regression weights on each energy component vary across different classes of reactivity.

The choice of benchmark set—and its division into these five families—is, of course, somewhat arbitrary. What we aim to demonstrate numerically below is that our RF/LR pipeline offers stable, transferable insights regardless of how one partitions the data. Increasing the size of the benchmark set and diversifying both the number and the sophistication of the linear models will likely yield even greater accuracy; in this sense, the present work should be viewed as a proof of concept.

All scripts used in this work are available at our GitHub repository (https://github.com/lstorchi/model_reaction_data).^33^

#### 2.1.4 Training the Linear Regression Models

For model training and optimization, we used two standard error metrics, MAE and MAPE, defined for model *i* over *N* reactions as follows

10 $$MAE\left(i\right)=\frac{1}{N}\sum_{j=1}^{N}\left|{\Delta E}_{j}^{CCSD\left(T\right)/CBS}-{\Delta E}_{j}^{M_{i}}\right|$$

11 $$MAPE\left(i\right)=\frac{1}{N}\sum_{j=1}^{N}\left|\frac{{\Delta E}_{j}^{CCSD\left(T\right)/CBS}-{\Delta E}_{j}^{M_{i}}}{{\Delta E}_{j}^{CCSD\left(T\right)/CBS}}\right|100$$

where Δ*E*_*j*_^CCSD(T)/CBS^ is the CCSD­(T)/CBS relative energy of each system *j*, ${\Delta E}_{j}^{M_{i}}$ is the approximated relative energy based on the weighted sum of energy decomposition terms for each system *j* and each model *i*, and *N* is the number of systems.

To obtain the final models for the various subsets we adopted a grid search. Specifically, for each functional and basis set, we started always building the LR­(FULL) model, that is the LR model built using all supersets. In this case, we start with *w* = 1and *b* = 0. Importantly, when *w* = 1and *b* = 0, Δ*E*_LR_ equals the reaction energy computed at the DFT level for a given functional/basis set combination. By construction, the model is thus designed to improve upon the baseline DFT values. Subsequently, we build the various superset models searching for the best results in terms of MAPE value using three different strategies as starting coefficients: (i) *w* = 1and *b* = 0; (ii) the ones coming from the LR­(FULL) model; (iii) the ones coming from an initial model converged using MAE as loss function. Similarly, during the grid search, we explored two optimization algorithms-BFGS and Nelder–Mead- and two distinct loss functions, MAE and MAPE. Ultimately, we selected the models that offered the best compromise between minimizing MAPE and maintaining a reasonable root mean squared error (RMSE), thereby ensuring both relative and absolute accuracy across the data set. Coefficients for all descriptors and combinations of functional and basis sets can be found in Table S1 (Supporting Information). It is important to underline here that our chosen approach involves ensuring that the final LR model expression accurately reflects a proper physical expression. Consequently, this methodology can occasionally lead to minor underfitting, as observed with the PBE/def2-SVP for the LARGE superset, or conversely, to some overfitting. To mitigate the overfitting issues, we adopted the previously mentioned L2 regularization. Specifically, this was applied to the PBE0/MINIX and PBE/MINIX SMALL models. Thus, the results presented for these two models utilize an L value (i.e., strength of the penalty) of 10.0 in the case of the PBE functional and 4.8 for the PBE0 one. Regarding the underfitting issue, which is apparent in both the PBE/def2-SVP LARGE and PBE/MINIX SMALL models, it is evidently quite mild. This can clearly be addressed in future work by employing more complex models instead of a purely LR one, or potentially by constructing new, more informative features from existing data.

#### 2.1.5 Performance of the Linear Regression Models

Figure 2 summarizes the MAPE values obtained for all LR models on the GMTKN55+LP14 benchmark set.

**Figure 2:**
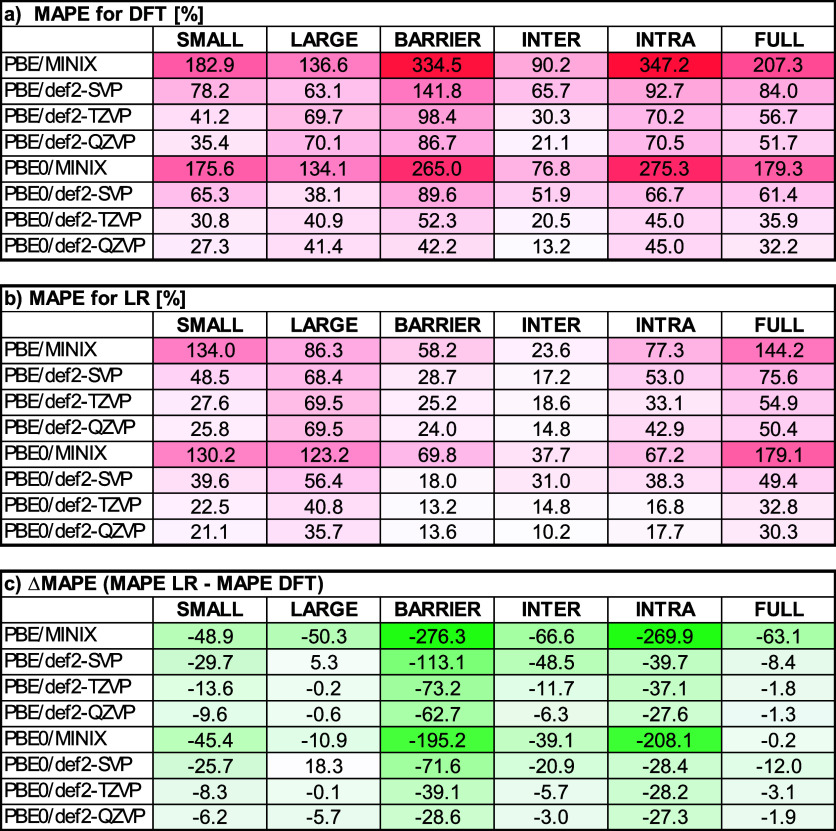
MAPE analysis of reaction-energy predictions across GMTKN55+LP14 supersets and global (FULL) data set. Panel (a) reports the MAPE of uncorrected DFT reaction energies for each combination of functional (PBE, PBE0) and basis set (MINIX, def2-SVP, def2-TZVP, def2-QZVP). Panel (b) shows the MAPE for the LR­(FULL) model or superset-specific models: LR­(SMALL), LR­(LARGE), LR­(BARRIER), LR­(INTER), LR­(INTRA). Panel (c) presents the relative change in MAPE (i.e., MAPE LR–MAPE DFT).

MAPE is a suitable loss function for this analysis because the reactive systems included in the benchmark vary widely in their energy scales. For example, the RG18 data set has an average absolute reaction energy of only 0.58 kcal/mol, while the DIPCS10 data set averages 654.26 kcal/mol.^31^ Expressing errors as percentages allows for meaningful comparisons across such disparate energy ranges by normalizing the error magnitude relative to the system’s intrinsic scale. To ensure the robustness of the models, we also report MAPE values separately for the training and test sets in Table S2 (Supporting Information). Although the final LR models were trained on the complete data set, the training/test split analysis confirms that the model performance remains stable and consistent, indicating the absence of significant overfitting.

Across all functionals, we observe a clear trend of decreasing MAPE as the basis set quality increases: for both uncorrected DFT (Figure 2a) and ML-corrected LR predictions (Figure 2b), MINIX exhibits the largest errors and def2-QZVP the smallest. LR models generally reduce MAPE across all functional/basis set combinations—not only for the specialized LR­(SUPERSET) models, but also for the global LR­(FULL)—demonstrating that even a single, general linear model can be used to enhance DFT accuracy.

The greatest error reductions occur with the smallest basis sets (e.g., PBE/MINIX sees a drop from 182.9% to 134.0% in SMALL; PBE0/MINIX from 175.6% to 130.2% in SMALL), reflecting the ability of the LR model to compensate for both BSIE and functional deficiencies. As the basis set expands and the intrinsic BSIE diminishes, the magnitude of the ML-driven improvement correspondingly decreases (e.g., PBE0/def2-QZVP SMALL improves only from 27.3% to 21.1%). Finally, as expected, specialized superset models consistently outperform the global LR­(FULL) correction: for barrier height reactions with PBE/def2-TZVP, the specialized LR­(BARRIER) achieves 25.2% MAPE versus 54.9% for LR­(FULL), underscoring the value of chemically tailored corrections. Of course, this raises the question of how to select the optimal LR model from our library for any given reaction. To solve this challenge, we employ the Random Forest-based selection protocol described below, which automatically assigns each system to the most appropriate superset model.

### 2.2 Random Forest-Guided Selection of the Optimal Linear Model

LR is a simple and effective model, particularly suited for data sets of limited size. Accordingly, using a dedicated LR model for each superset is expected to yield better accuracy than a single, general model, as demonstrated numerically in the previous sections. However, maintaining multiple models poses challenges in terms of robustness and practical deployment. To address this trade-off between specialization and generalization, we built an RF classifier as a preliminary step (Figure 3).

**Figure 3:**
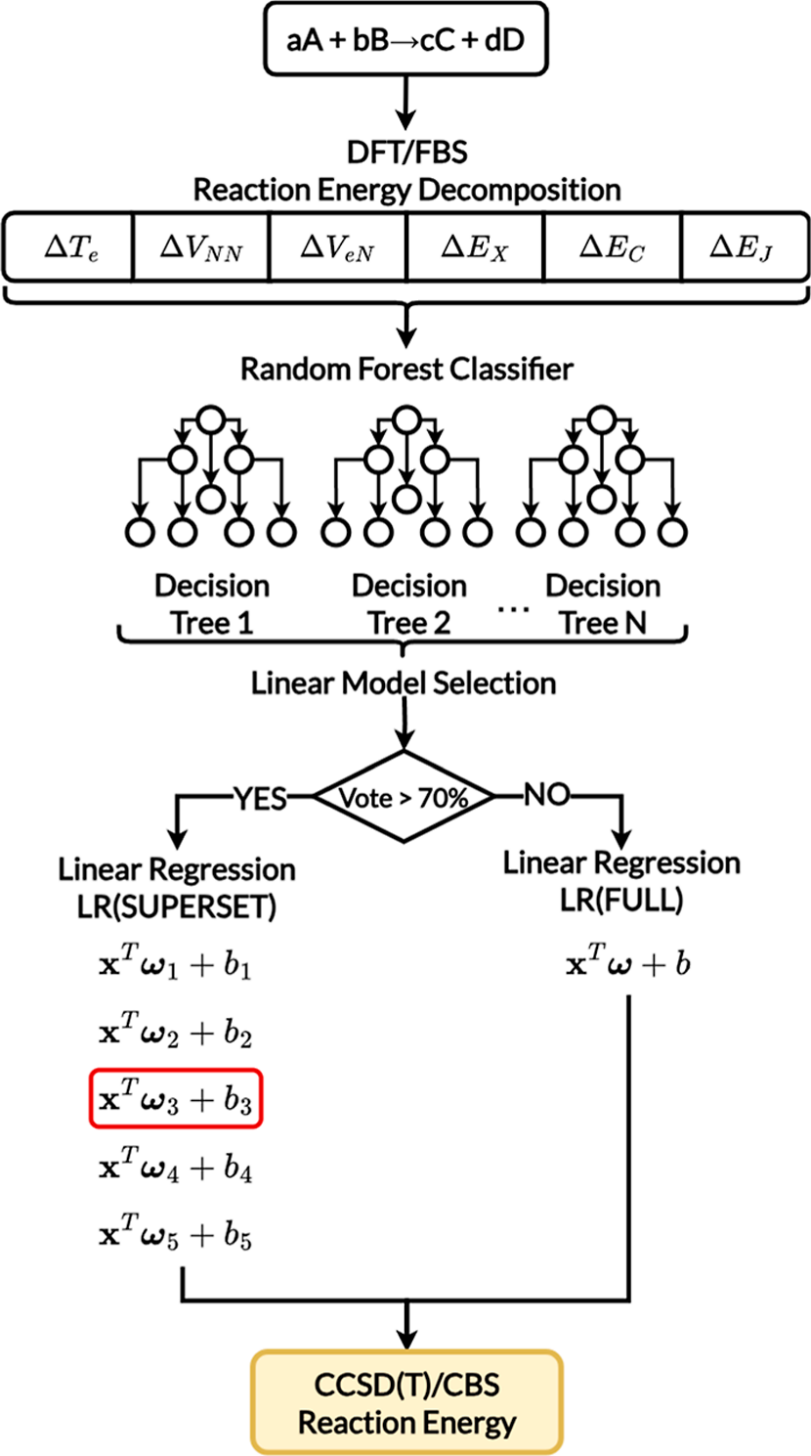
Coupled Random Forest with Linear Models: first, a linear model is trained for each type of chemical problem and for the complete data set (Full). Afterward, for each system, a random forest classifier selects the most appropriate model to use based on the energy decomposition terms. If the voting made by the classifier is lower than 70%, the LR­(FULL) model is used to approximate the exact final energy.

Trained using the same features and data sets as the LR models, the RF model assigns a reaction to one of the predefined supersets. Based on this classification, the corresponding specialized LR model is then used to predict the relative energy. To enhance reliability, the RF model leverages the physically meaningful nature of the energy decomposition descriptors for classification. If the classification confidence (based on majority vote) exceeds a 70% threshold, the appropriate superset-specific model is used; otherwise, the prediction defaults to the global LR­(FULL) model. This hybrid scheme combines the predictive accuracy of specialized models with the flexibility and robustness of a unified framework, offering a practical and high-performing solution.

For each combination of functional and basis set, a separate RF model was trained using 80% of the available data for training and the remaining 20% for testing. The model hyperparameters (specifically, the number of decision trees and the maximum depth of each tree) were optimized via a basic grid search, selecting the configuration that achieved the highest test set accuracy. The final models, detailed in Table S3 (Supporting Information), included various numbers of trees depending on the best-performing setup.

In some cases, the optimal configuration involved allowing trees to grow until all leaves were pure, meaning that each leaf contained samples from only a single class. This approach minimizes Gini impurity^34^ by continuing to split nodes if it improves classification purity. The RF models generally performed very well, achieving an overall classification accuracy (i.e., a final accuracy over the entire data set) above 93% in most cases. We consider this to be a solid compromise between complexity and predictive power.

RF and LR models are available as pickles at https://github.com/lstorchi/model_reaction_data.^35^

### 2.3 Overall Performance Evaluation

As discussed above, the error associated with each functional/basis set combination relative to CCSD­(T)/CBS decreases with increasing basis set size. Similarly, the inclusion of Hartree–Fock exchange (as in hybrid functionals) generally reduces the inherent DFT error. Figure 4 illustrates this trend by showing the correlation between predicted and reference values for the GMTKN55 data set, alongside the MAPE, for two representative cases: (i) a GGA functional with a small basis set PBE/MINIX (MAPE = 209.0%) and (ii) a hybrid GGA functional with a large basis set PBE0/def2-QZVP (MAPE = 32.4%).

**Figure 4:**
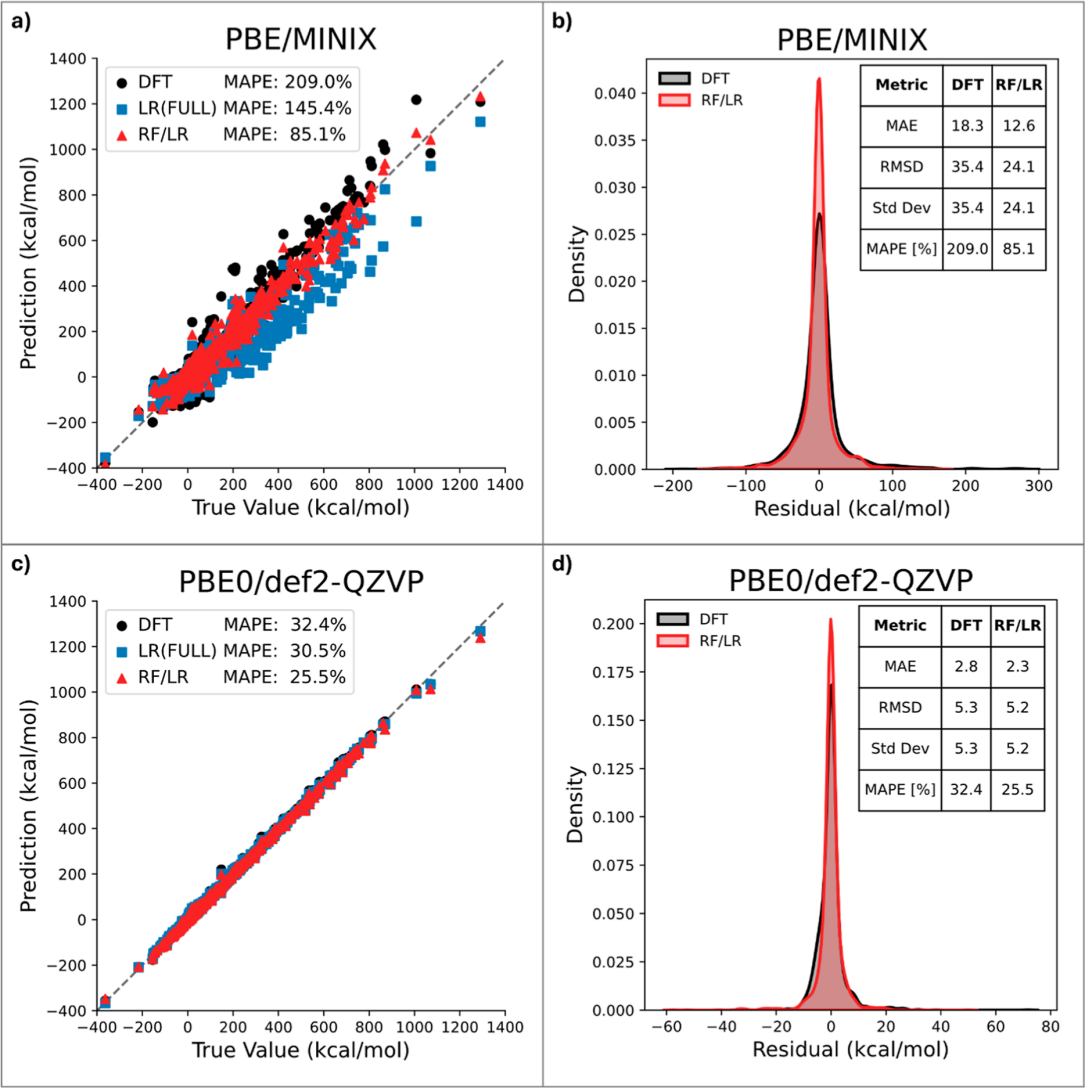
Comparison of predicted versus reference relative energies for the GMTKN55 data set using PBE/MINIX (a) and PBE0/def2-QZVP (c). Results are shown for plain DFT alongside predictions from the LR­(FULL) and RF/LR models. Panels (b) and (d) display the distribution of residuals for PBE/MINIX and PBE0/def2-QZVP, respectively, with key metrics (MAE, RMSD, standard deviation, MAPE) illustrating the improved accuracy achieved by the RF/LR model in both cases.

Upon applying the LR­(FULL) model, the MAPE for PBE/MINIX drops to 145.4%, and further decreases to 85.1% with the RF-guided model. This represents a reduction of more than 50% in the original error, achieved at negligible additional computational cost (Figure 4a). Additionally, Figure 4b shows the distribution of residuals for both DFT and RF/LR. Compared to plain DFT (MAE = 18.3, RMSD = 35.4, MAPE = 209.0%), the RF/LR pipeline achieves lower errors (MAE = 12.6, RMSD = 24.1, MAPE = 85.1%) and a reduced error spread (σ = 24.1 vs 35.4). This indicates not only a systematic increase in accuracy but also a tighter distribution of residuals, with errors more strongly centered around zero.

For PBE0/def2-QZVP, the LR­(FULL) and RF/LR models yield MAPEs of 30.5% and 25.5%, respectively (Figure 4c). While the absolute improvement is smaller in this case, it demonstrates that our approach can even improve high-level DFT results with a large basis. The distribution of residuals shown in Figure 4d further supports these findings. Although the uncorrected DFT results already exhibit relatively low errors (MAE = 2.8, RMSD = 5.3, MAPE = 32.4%), the RF/LR pipeline achieves a further reduction (MAE = 2.3, RMSD = 5.2, MAPE = 25.5%) while maintaining a nearly identical error spread (σ = 5.2 vs 5.3). This demonstrates that the model remains robust, with RF/LR providing a subtle improvement without compromising prediction reliability.

To generalize these findings across all our models, Figure 5a reports the final MAPE values over the entire GMTKN55+LP14 database for three approaches: raw DFT predictions, the global LR­(FULL) model, and the RF-guided LR pipeline (RF/LR). Several clear trends emerge.

I. As discussed above, uncorrected DFT errors decrease steadily as the basis set improves (MINIX → def2-QZVP) and when Hartree–Fock exchange is added (PBE → PBE0). For example, PBE/MINIX starts with a MAPE of 207.3%, whereas PBE0/def2-QZVP achieves 32.2%.
II. Applying a single, universal linear-regression model reduces the DFT error in almost every case. The most dramatic reduction (−63.1 percentage points) occurs for PBE/MINIX (from 207.3% to 144.2%), showing that the largest gains appear for the smallest basis sets, where BSIE are the largest. Notably, in some cases, the improvement is almost negligible, reflecting the limitations of a one-size-fits-all model when the baseline error is already low.
III. By routing each reaction to its optimal superset-specific linear model using our RF/LR approach, errors shrink even further. PBE/MINIX MAPE falls to 84.4% (a 122.9 percentage point reduction), and PBE0/MINIX to 101.9% (a 77.4 percentage point reduction). Even high–accuracy combinations such as PBE0/def2-QZVP benefit, decreasing from 32.0% to 25.3% (a 6.7 percentage point reduction).

**Figure 5:**
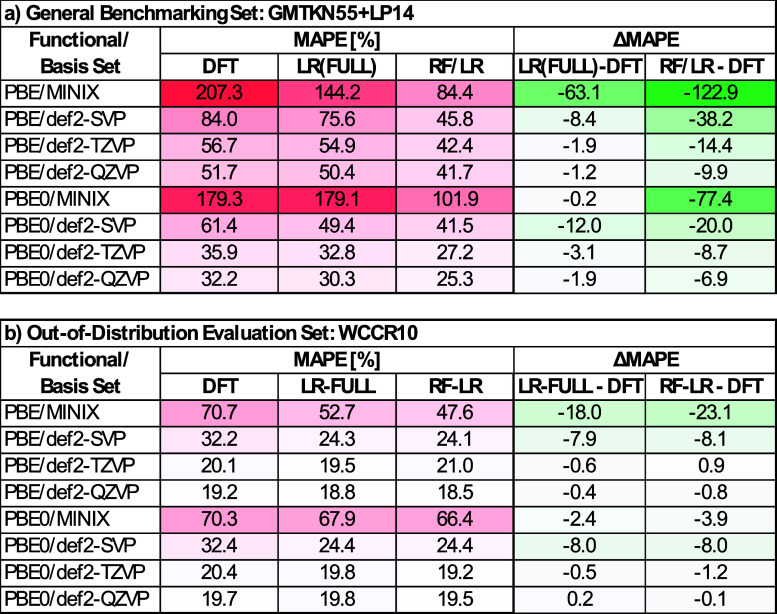
MAPE for different functionals and basis sets, and ΔMAPE comparing raw DFT results with LR models: LR­(FULL) (linear regression trained on the full data set) and RF/LR (linear regression guided by random forest feature selection). (a) Results on the GMTKN55+LP14 benchmark set. (b) Results on the WCCR10 data set used for out-of-distribution evaluation (this is the only set considered in this work containing transition metals, which are not included in the training set). LR models consistently reduce errors compared to uncorrected DFT, especially in low-cost basis sets. The worst-performing DFT combinations (highlighted in red) see the largest improvements from ML correction.

These results demonstrate that simple, interpretable LR models—especially when combined with RF-based model selection—can improve the accuracy of low-cost DFT calculations with no additional computational cost.

Finally, to assess the robustness of our approach beyond the chemical space represented in GMTKN55+LP14, we next turn to an out-of-distribution evaluation using the WCCR10 benchmark^36^—comprised of large, cationic transition-metal complexes that were entirely absent from our training set. This stress test reveals the ability of our ML-LR framework to extrapolate reliably when confronted with novel chemistries.

Remarkably, both the global LR­(FULL) model and the RF/LR pipeline maintain or improve upon raw DFT accuracy for every functional and basis-set combination. For example, PBE/MINIX MAPE falls from 70.7% to 52.7% with LR­(FULL), and further to 47.6% with RF/LR. Similarly, PBE0/MINIX drops from 70.3% to 67.9% and then to 66.4%. The highest-quality method, PBE0/def2-QZVP, maintains its accuracy (19.7% → 19.8% → 19.5%).

This performance demonstrates that, even in the absence of any transition-metal-specific LR model, the RF classifier reliably selects the most suitable model—typically LR­(FULL)—avoiding catastrophic failures. Unlike overparametrized models that can produce large errors when extrapolating, our hybrid approach combines interpretable linear corrections—grounded in physical energy components—with an adaptable RF selector. As additional specialized models (e.g., transition-metal-focused LR variants) are developed, the RF/LR pipeline will automatically incorporate them, ensuring continuous, modular improvement in predictive accuracy across an ever-expanding chemical landscape.

#### 2.3.1 Interpretable Models

To comprehend the factors influencing our predictions, a two-tiered model analysis can be undertaken. For the RF, which serves as the high-level selection mechanism, a Permutation Feature Importance analysis can be employed.^20,37^ This method is particularly suitable because RF, as a tree-based ensemble, is insensitive to feature scaling, thereby enabling an evaluation of the features it prioritizes when selecting the optimal linear model. In contrast, for the LR models, the features are not normalized, and hence direct comparisons of coefficient magnitudes to assess feature importance might be misleading. Instead, we can interpret the coefficient trends across the various models, each associated with a specific functional and basis set.

Specifically, our LR models aim to determine optimal weights for different energy components, thereby improving prediction accuracy. Importantly, the difference between the LR prediction and that of the underlying DFT calculation vanishes when the model intercept is set to 0 and all other coefficients equal to 1. Examining the coefficients reported in Table S1 (Supporting Information), we observe that they tend to approach 1.0 as the basis set size increases (e.g., from MINIX to def2-SVP, def2-TZVP, and def2-QZVP). This convergence originates from the fact that larger basis sets lead to smaller BSIEs and hence yield more accurate underlying calculations, thus requiring less correction from the LR model. Exceptions exist, however, and convergence is not always monotonic. For example, in the SMALL superset, the coefficients obtained with the def2-SVP basis set deviate more strongly from 1.0 than those obtained with the smaller MINIX basis set. Nevertheless, the overall trend is well maintained in most cases.

The intercept term in a linear model instead represents a constant, systematic offset. An intercept different from zero implies that the LR model predicts a difference between direct and inverse reaction energies (aside from the trivial change of sign). Ideally, the intercept should be as close to zero as possible. A large intercept, by contrast, indicates a notable baseline error in the original calculations. Consistently, as the basis set size increases from MINIX to def2-QZVP, the magnitude of the intercept generally decreases and approaches zero, although exceptions remain. This behavior suggests that larger basis sets produce a more accurate baseline calculation, as expected. For example, in the LARGE superset using the PBE functional, the intercept decreases from 2.15 kcal/mol with the MINIX basis set to 0 with the TZVP basis set.

Importantly, while an intercept of 2.15 kcal/mol may appear especially large—potentially undermining confidence in model predictions when the PBE/MINIX basis set is used—it should be emphasized that the intercept is always orders of magnitude smaller than the inherent error of both the LR models and the underlying DFT calculations, as measured in terms of MAE. For example, for PBE/MINIX in the SMALL superset, the MAE of the LR model is about 25.2 kcal/mol, while that of the underlying DFT calculation is 28.7 kcal/mol. Hence, the error associated with a nonzero intercept accounts for only a minor fraction of the overall model error. Overall, intercepts obtained with the PBE0 functional are consistently closer to zero than those obtained with PBE, particularly with larger basis sets. For instance, with the def2-TZVP basis set, most PBE0 models have intercepts very close to zero (e.g., 0.0001 kcal/mol for BARRIER and −0.00008 kcal/mol for SMALL). This indicates that PBE0 provides a more reliable starting point with less systematic error than PBE.

To identify the features most relevant to the Random Forest model, we employ the Permutation Feature Importance analysis. This method is preferred over the default Gini importance^37^ due to its more reliable assessment of a feature’s predictive power on unseen data. The analysis is performed on both training and test sets to identify which features offer robust and generalizable predictive value. To ensure statistical stability, importance scores were averaged over 30 permutation rounds, and multiple classification metrics-accuracy, F1 score, precision, and recall-were considered. The results were reported in Figure S5. Across all functionals and basis sets, Δ*E*_*X*_ and Δ*E*_*C*_ are consistently ranked as the most significant features, while Δ*T*_*e*_ follows as the third most significant feature. Increasing the basis set size primarily reduces the importance of minor features on the test set.

## 3 Conclusions

In this study, we have demonstrated that simple, physically grounded machine-learning models can significantly increase the accuracy of DFT reaction-energy predictions while preserving interpretability. By decomposing reaction energies into seven fundamental components—kinetic, nuclear repulsion, electron–nuclear attraction, exchange, Coulomb repulsion, correlation, and dispersion—and training linear-regression models on these descriptors, we exploit intrinsic error cancellation and focus exclusively on chemically meaningful differences. Our global model LR­(FULL) yields significant reductions in MAPE across all functional/basis set combinations, with the most pronounced gains observed for small basis sets where basis set incompleteness errors are the largest.

To tailor corrections to distinct chemical regimes, we developed superset-specific LR models. A RF classifier then assigns each reaction to its optimal model—the RF/LR pipeline—achieving further error reductions of up to 122.9 percentage points. This two-stage strategy leverages the predictive accuracy of specialized corrections within the robustness of a unified framework, eliminating the need for any single model to compromise between specificity and generalizability.

We subjected our framework to an out-of-distribution stress test using the WCCR10 transition-metal benchmark. Despite the absence of any metal complexes in training, our RF/LR pipeline maintained or improved upon raw DFT errors across all methods, indicating stable extrapolation and graceful fallback to the global model when specialized submodels are unavailable. This stands in stark contrast to ANNs, which often suffer accuracy collapse under extrapolative conditions due to their opaque, overparameterized nature.

From a practical standpoint, our approach is highly efficient, requiring only low-cost single-point DFT calculations followed by lightweight ML postprocessing. It also provides chemically interpretable insights into the sources of error and supports flexible expansion through its modular design—enabling the incorporation of new specialized models as additional data become available.

Finally, the linearity of our regression offers a unique conceptual advantage: once the optimal weights are determined from relative-energy training, the same parameters can, in principle, be applied directly to absolute energy corrections. While this possibility was not explored in the present work, it opens an interesting avenue for future research. In particular, one could envision combining relative and absolute energy data during training to further enhance model robustness and flexibility. Additional future efforts will expand the model library to include organometallic and solid–state reactions and explore hybrid schemes that integrate nonlinear corrections, where necessary. We believe that this framework, which combines accuracy, transparency, and adaptability, offers a promising route toward increasingly reliable quantum chemistry simulations.

4

Coefficients for all regression models, MAPE for the training and testing sets, RF structure, and correlation plots for all combinations of functional and basis set for the GMKTN55, LP14, and WCCR10 can be found in the Supporting Information.

All codes developed for this work are available at https://github.com/lstorchi/model_reaction_data and a versioned archive is available in Zenodo.^29,33,35^

## Supplementary Material


