# Supplementary material for: Transferable and Transparent Energy Decomposition-Based Machine Learning Models for Computing Accurate Reaction Energetics
Source: J Chem Theory Comput. 2025 Oct 29;21(21):10853–62. doi: 10.1021/acs.jctc.5c01184 (PMC12613314; doi:10.1021/acs.jctc.5c01184)
Supplement: Supplementary file 1 [file ct5c01184_si_001.pdf]

Supporting Information for:

Transferable and Transparent Energy Decomposition-based Machine Learning Models for Computing Accurate Reaction Energetics

Carlos R. Jacinto-Mejía,[a] Loriano Storchi,[b] and Giovanni Bistoni*[a]*

[a] Carlos R. Jacinto-Mejía, G. Bistoni
Department of Chemistry, Biology and Biotechnology, University of Perugia, 06123 Perugia, Italy
*Email: giovanni.bistoni@unipg.it
[b] L. Storchi
Dipartimento di Farmacia, Universita' G. d'Annunzio, 66100 Chieti-Pescara, Italy
*Email: loriano@storchi.org

ORCID IDs
Carlos R. Jacinto-Mejía, https://orcid.org/0000-0002-3554-9699
L. Storchi, https://orcid.org/0000-0001-5021-7759
G. Bistoni, https://orcid.org/0000-0003-4849-1323

Contents

A. Linear Regression Coefficients 1
B. Training and Testing Mean Absolute Percentage Errors 4
C. Random Forest Classifiers Structure 5
D. Correlation Plots for Datasets in the GMTKN55, LP14, and WCCR10 datasets 6
E. Permutation Feature Importance Analysis 11
References 12

A. Linear Regression Coefficients

Table S1. Linear regression coefficients for all combinations of functional and basis set, for all descriptors used in this study. The columns represent the dataset used to train the models.

Functional/ Basis Set	Coefficient	SMALL	LARGE	BARRIER	INTER	INTRA	FULL
PBE/ MINIX	Intercept	-0.000907143028	2.15170068664670	1.54630623332041	-0.05303839589929	0.25964171291397	0.32420785045572
	Te	0.9679428601	0.96945857576363	0.40619305574138	0.43969006494114	0.45632215291927	0.85591622098159
	V_NN	0.9876947165	0.96695539110390	0.35459515109469	0.45822686101985	0.42991377844806	0.84568002574337
	V_eN	0.9925019853	0.97753578543759	0.35294961488818	0.45845887818712	0.43083828750239	0.85333622335001
	EX	1.087151716	1.20656297120504	0.92191033633986	0.46095988767952	1.01202476731213	1.13906682788043
	EC	0.3190155464	-0.17657342123791	-0.55653608909749	0.26030853492426	-0.23274023091840	-0.60484144401138
	EJ	0.997298835	0.98811220082475	0.35134964376389	0.45868797022495	0.43175822375251	0.86098939438514
PBE/ def2-SVP	Intercept	0.22603735049623	-0.00070720743619	0.32784579425690	-0.22254900017550	0.14191464628997	-0.00070720743887
	Te	0.87443328241881	0.99585392382802	0.88222373599064	0.51686839290414	0.78353130594766	0.99585392396669
	V_NN	0.92153519816778	1.00426593204380	0.86809857152536	0.51278442569035	0.77903391179613	1.00426589047757
	V_eN	0.92115631687530	1.00088412034127	0.87442330884228	0.51192124922960	0.77982773536149	1.00088420301447
	EX	1.01390269910678	1.00096501444840	1.19821418331245	0.65718496791062	0.99360001755318	1.00096501445776
	EC	0.82195245840534	0.99942703274318	-0.38870810505760	0.40741285768322	0.00752802118494	0.99942703278608
	EJ	0.92080030075405	0.99750736447658	0.88060160857278	0.51105783119181	0.78059825811282	0.99750732314979
PBE/ def2-TZVP	Intercept	-0.12423226984426	-0.00000000000001	0.17864028527334	-0.20207517534908	0.06368584825341	-0.20045209418301
	Te	0.95378947567273	0.99999999999970	1.00750148027942	0.95195420945407	0.79828242180759	0.94489474199009
	V_NN	0.98276842678723	0.99999995056378	0.96712685559159	0.99714308139494	0.85160692919367	0.99995851925829
	V_eN	0.97591814979622	1.00000006336373	0.97153904758147	0.99736070132042	0.85024417253835	0.99986544357174
	EX	0.79671076500391	0.99999999999985	1.21871770634353	1.00809516485743	0.83956922579142	1.01134625698993
	EC	1.18905835391840	1.00000000000004	0.00132763530164	0.79296528723002	0.63250999916997	0.79273230112539
	EJ	0.96907076831109	0.99999995055677	0.97583773490897	0.99757672275341	0.84887574812328	0.99977195942126

Functional/ Basis Set	Coefficient	SMALL	LARGE	BARRIER	INTER	INTRA	FULL
PBE/ def2-QZVP	Intercept	0.17102093031502	-0.00000000000002	0.32663091723934	-0.20862927744630	0.05615071928214	-0.18714342813163
	Te	0.96669705364961	0.99999999999883	1.01710909136139	0.96040803973322	1.00420679247304	0.95413926113392
	V_NN	0.99424402621080	0.99999990573554	0.98141673948829	1.00007718523833	1.00135097306386	0.99994456812675
	V_eN	0.98965680800064	1.00000018386940	0.98417100777923	0.99982228877950	0.99999293823443	0.99982230980672
	EX	0.85901361553352	0.99999999999983	1.20329606955835	1.00792065420547	0.99947507805333	1.01257989015550
	EC	1.01927944020531	1.00000000000013	0.09725770432204	0.83600302733212	0.65162901107682	0.79995624635849
	EJ	0.98504619523616	0.99999990572213	0.98683735763737	0.99956689996050	0.99861522502586	0.99969953098630
PBE0/ MINIX	Intercept	-0.000421206925	0.01633817561552	1.48004515254577	-0.17561481686245	0.32676196578053	-0.00000000000579
	Te	0.949111038778	0.96412122376201	0.71023913112113	0.33941943123032	0.46624466658032	0.99999999989874
	V_NN	0.982550863521	0.98863728124358	0.67007722588379	0.33057804638306	0.44379729166471	1.00000017404039
	V_eN	0.995476390674	0.99200906605990	0.67647764618349	0.33240708009459	0.44466168868623	0.99999965209671
	EX	1.09135492836	0.86673230264038	1.43388573344642	0.69367400622216	1.18058481383566	1.00000000001212
	EC	1.276919726609	0.89798199729132	-1.03275092791678	0.17127188452339	-0.36662562241488	0.99999999999213
	EJ	1.008588781213	0.99537989570493	0.68275419301091	0.33423610753208	0.44552061714290	1.00000017393777
PBE0/ def2-SVP	Intercept	-0.29505452304737	-0.08667281017853	-0.04550362239351	-0.08667281017889	0.16959259838547	-0.08667281017881
	Te	0.87533090538602	0.98302477400871	0.84648829298249	0.98302477401238	0.62738580045805	0.98302477401047
	V_NN	0.94761818720312	0.99993095547436	0.84334877352192	0.99993099998773	0.66542377374184	0.99993099555447
	V_eN	0.94553506446381	0.99999537106958	0.84744626423399	0.99999530080961	0.66518527604048	0.99999531339267
	EX	0.98186071568526	1.13455424180284	1.14553998068081	1.13455424180511	0.74907642141736	1.13455424180490
	EC	0.93919205455923	0.79581461966964	0.01923240087714	0.79581461967089	0.69299542723925	0.79581461967055
	EJ	0.94343444737576	1.00006004323487	0.85143487622490	1.00006008776133	0.66495156452565	1.00006008332542

Functional/ Basis Set	Coefficient	SMALL	LARGE	BARRIER	INTER	INTRA	FULL
PBE0/ def2-TZVP	Intercept	-0.00008559197715	0.00000000000006	0.00016443753495	0.00056714370279	0.00673454615101	0.00072255951206
	Te	1.00498620473016	0.99999999999974	1.05531669012776	0.98199881131133	0.96425951099040	0.98341690110174
	V_NN	1.00481274278916	0.99999998051163	0.99742686812412	1.00014057421641	1.00184631454205	0.99998704756095
	V_eN	1.00141372891143	1.00000003898204	1.00051643853896	1.00008566752278	1.00062144185756	0.99997823559044
	EX	0.90945631065345	0.99999999999947	1.19939581935598	0.96745024009058	0.84281375761535	1.03753778050470
	EC	0.99192537270358	0.99999999999976	0.30507446843647	0.83908808135291	0.91853417934942	0.81019180668129
	EJ	0.99800714170399	0.99999998050710	1.00352654260833	1.00003034932011	0.99940030090374	0.99996891806633
PBE0/ def2-QZVP	Intercept	-0.001425194021	0.35330834165958	0.00080981789653	0.00056032268851	0.00478184398781	0.00046064417836
	Te	0.992938687169	0.96734311018766	1.07806983524331	0.99600347616853	0.98590033661262	0.99493760574221
	V_NN	1.002021785488	0.99978464469887	1.00603739067212	0.99999092034465	1.00291130436067	0.99996363712945
	V_eN	1.00142216934	1.00000017028176	1.00650766872987	0.99999960013921	1.00107590315516	0.99997335930597
	EX	0.977413589072	0.90399798631513	1.28483724831420	0.95205739974656	0.83142931403300	1.00949470868087
	EC	0.993606025026	1.03202395481445	0.49211756448041	0.91754496476477	1.02503417665514	0.88508429715019
	EJ	1.000822081838	1.00021523781786	1.00694676263877	1.00000751335195	0.99924431782541	0.99998235903668

B. Training and Testing Mean Absolute Percentage Errors

Table S2. Mean absolute percentage error (MAPE) for training and testing sets for all combinations of functionals and basis set. The columns indicate the dataset used to train the model.

Dataset	BARRIER	INTRA	SMALL	INTER	LARGE	FULL
**PBE/MINIX**						
Training set	0.818	0.907	0.693	0.401	0.786	0.914
Test set	0.731	0.694	0.545	0.464	1.609	1.065
**PBE/ def 2-SVP**						
Training set	0.346	0.509	0.373	0.329	0.812	0.641
Test set	0.252	0.658	1.401	0.334	0.768	1.183
**PBE/ def 2-TZVP**						
Training set	0.255	0.331	0.279	0.154	0.719	0.461
Test set	0.188	0.329	0.403	0.128	0.562	0.914
**PBE/ def 2-QZVP**						
Training set	0.276	0.448	0.246	0.181	0.713	0.464
Test set	0.145	0.322	0.495	0.166	0.592	0.929
**PBE0/MINIX**						
Training set	0.722	0.670	1.154	0.268	1.357	1.514
Test set	0.598	0.725	2.840	0.303	1.269	3.362
**PBE0/ def 2-SVP**						
Training set	0.187	0.326	0.303	0.226	0.538	0.413
Test set	0.160	0.352	0.930	0.278	0.596	0.735
**PBE0/ def 2-TZVP**						
Training set	0.138	0.170	0.206	0.158	0.404	0.291
Test set	0.105	0.170	0.460	0.134	0.421	0.537
**PBE0/ def 2-QZVP**						
Training set	0.157	0.186	0.232	0.102	0.401	0.253
Test set	0.097	0.140	0.349	0.094	0.451	0.580

C. Random Forest Classifiers Structure

Table S3. Random forest classifiers structure for all combinations of functional and basis set. For more details, refer to the main text.

Functional/ Basis Set	Number of Decision Trees	Maximum Depth of each Tree	Training Set Accuracy	Test Set Accuracy
PBE/ MINIX	20	15	1.00	0.78
PBE/ def2-SVP	70	10	0.97	0.78
PBE/def2-TZVP	140	Expanded until all leaves are "pure"	1.00	0.78
PBE/def2-QZVP	80	15	1.00	0.80
PBE0/MINIX	40	Expanded until all leaves are "pure"	1.00	0.78
PBE0/def2-SVP	40	Expanded until all leaves are "pure"	1.00	0.80
PBE0/def2-TZVP	180	Expanded until all leaves are "pure"	1.00	0.79
PBE0/def2-QZVP	130	Expanded until all leaves are "pure"	1.00	0.82

D. Correlation Plots for Datasets in the GMTKN55, LP14, and WCCR10 datasets

The data used in this work to train the Linear Regression and Random Forest models was a combination of the General Main Group Thermochemistry, Kinetics, and Non-Covalent Interactions (GMTKN55) dataset[1] and Frustrated Lewis Pairs and Classical Lewis Adducts (LP14) dataset[2]. Additionally, for a rigorous out of distribution test, we employed the Ligand Dissociation Energies of Large Cationic Transition Metal Complexes (WCCR10) dataset[3]. Figures S1, S2, and S3 show correlation plots for these datasets between reference values (mostly CCSD(T)/CBS) and predictions made using standard DFT, out LR(FULL), and our RF/LR model. Additionally, for each plot, the MAPE for each case is shown.

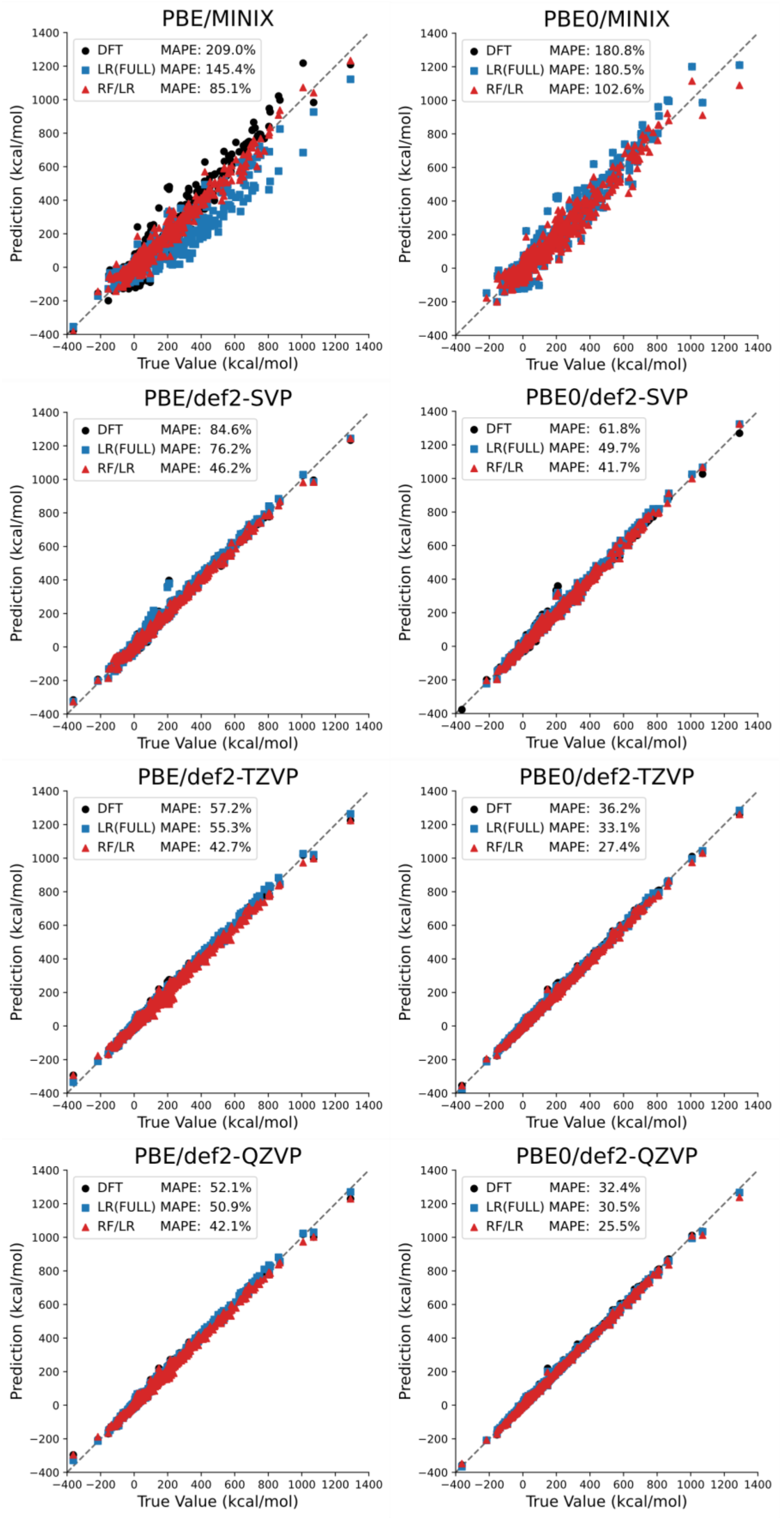

Figure S1. Correlation plots for GMTKN55.

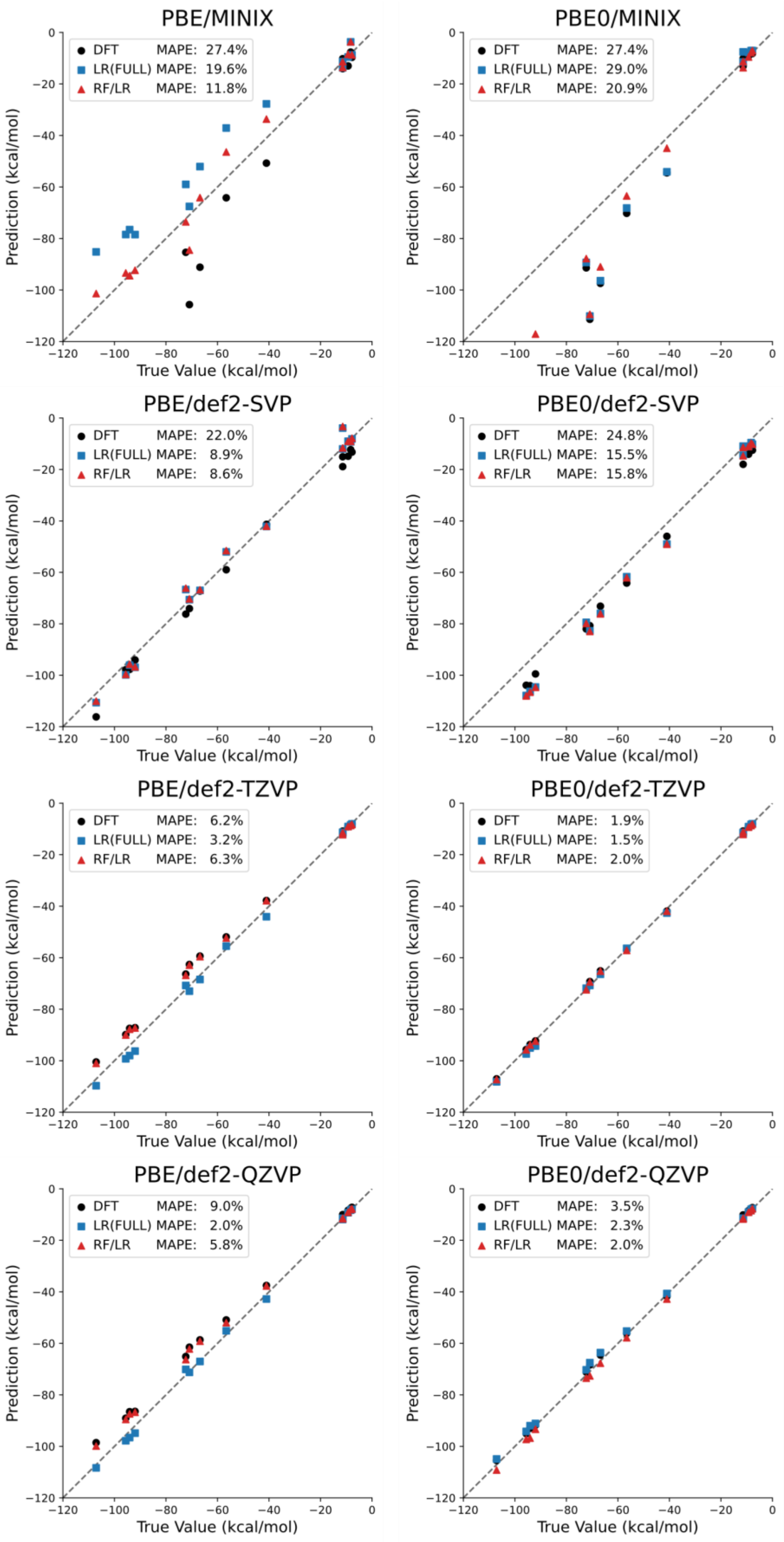

Figure S2. Correlation plots for LP14.

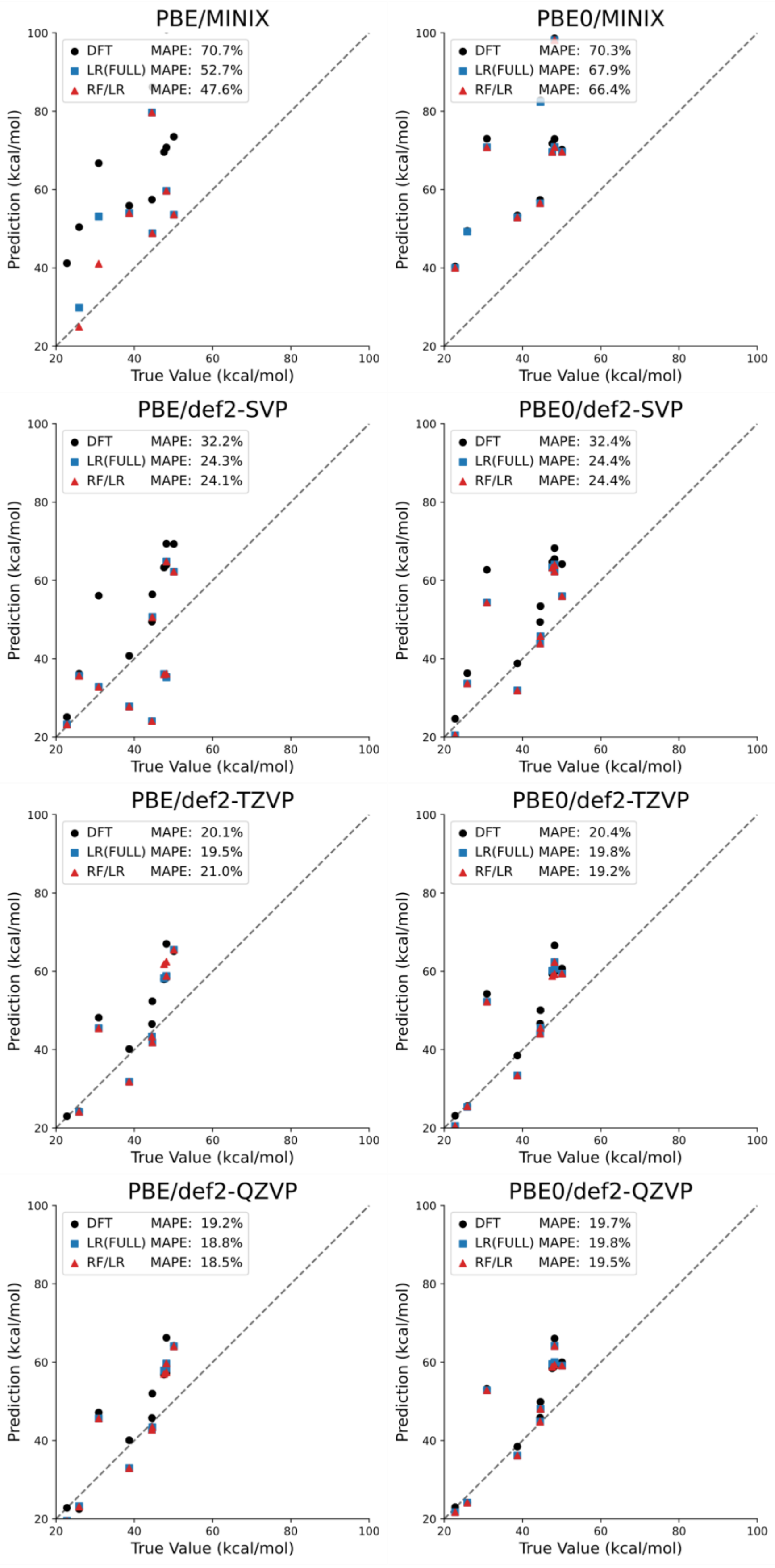

Figure S3. Correlation plots for WCCR10.

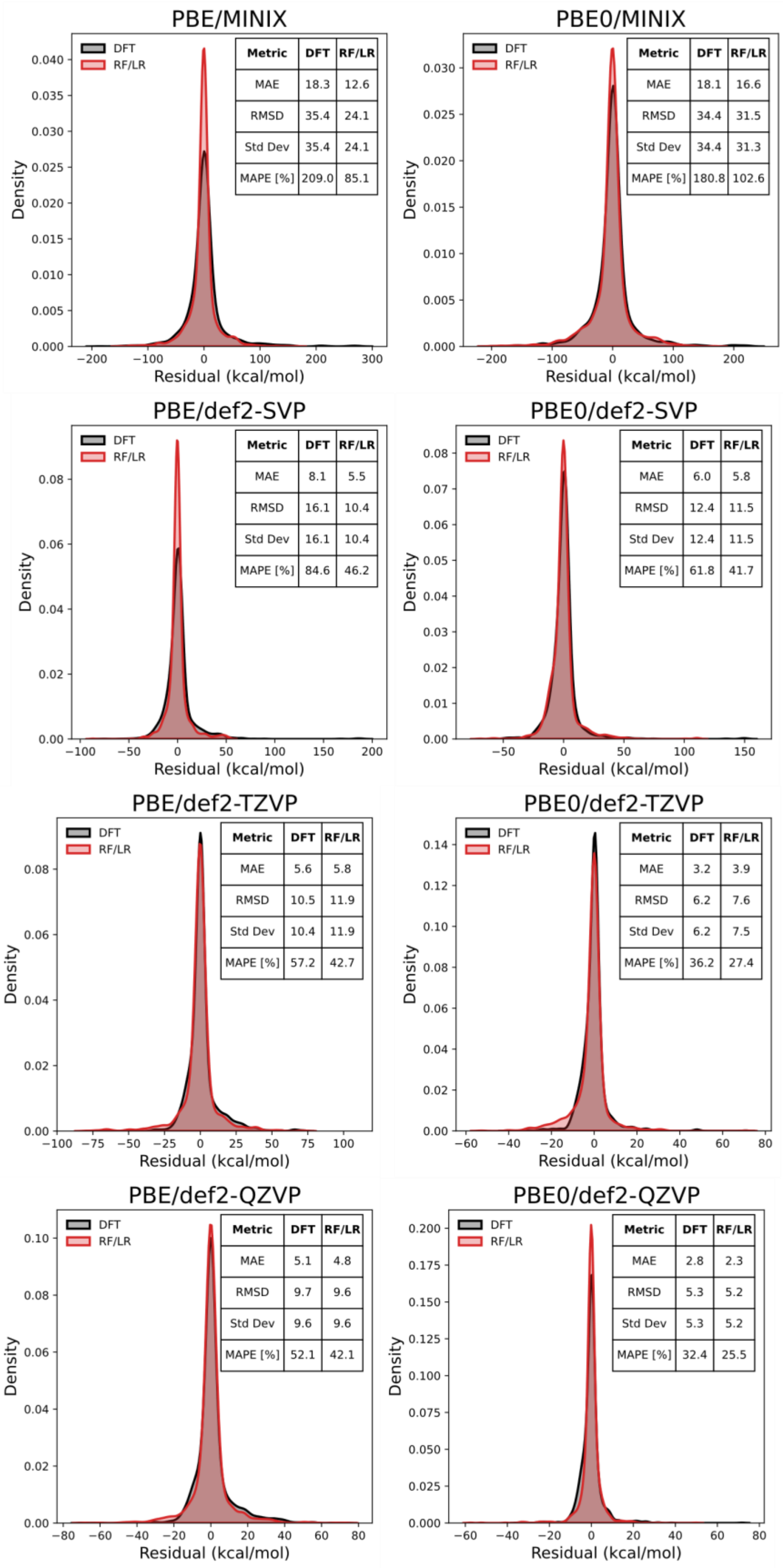

Figure S4. Distribution of residuals for all combinations of functionals and basis sets

E. Permutation Feature Importance Analysis

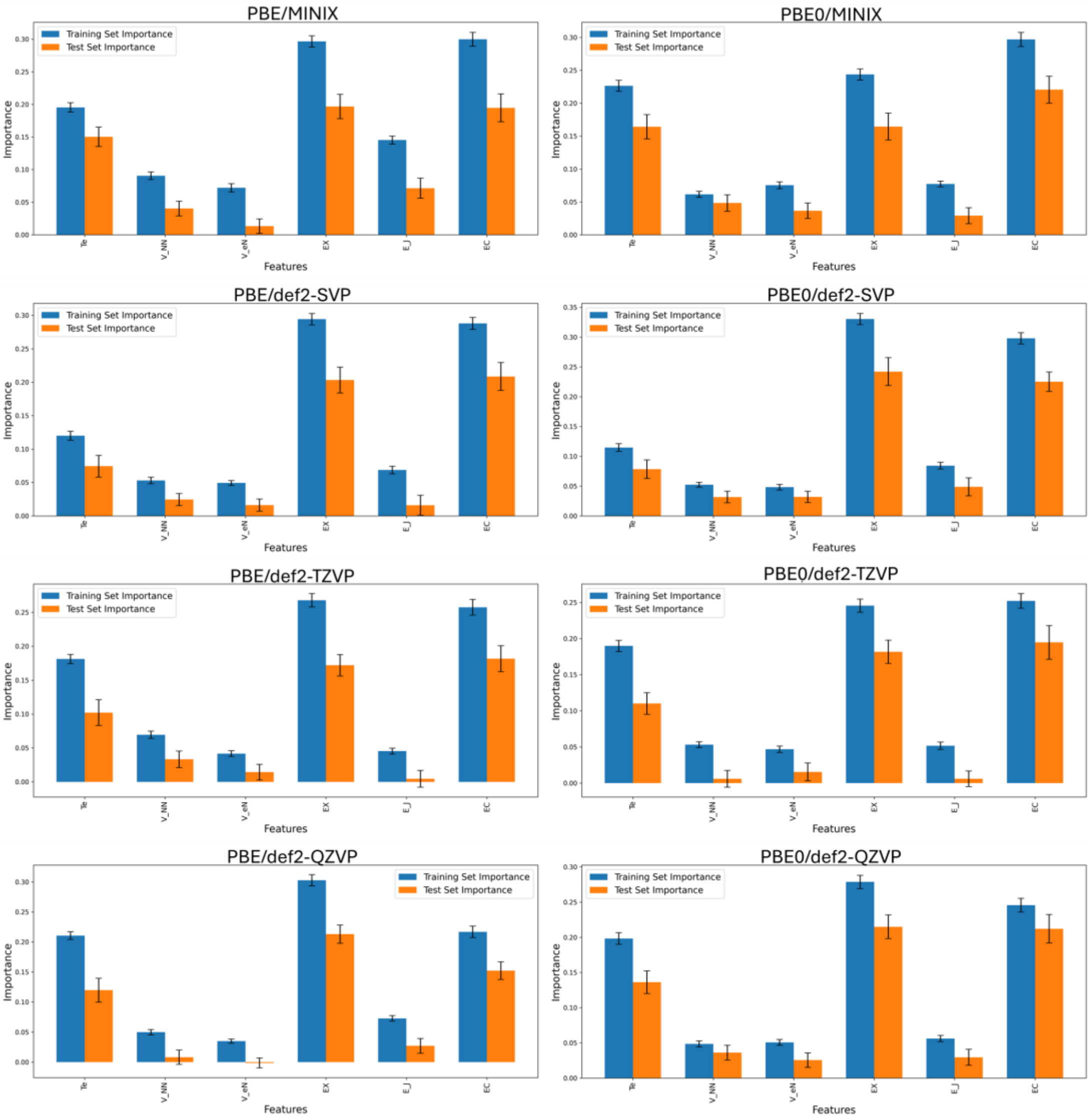

Figure S5. Results of the permutation feature importance analysis of the random forest models for all combinations of functionals and basis sets

References

[1] L. Goerigk, A. Hansen, C. Bauer, S. Ehrlich, A. Najibi, and S. Grimme, "A look at the density functional theory zoo with the advanced GMTKN55 database for general main group thermochemistry, kinetics and noncovalent interactions," *Physical Chemistry Chemical Physics*, vol. 19, no. 48, pp. 32184–32215, 2017, doi: 10.1039/c7cp04913g.

[2] G. Bistoni, A. A. Auer, and F. Neese, "Understanding the Role of Dispersion in Frustrated Lewis Pairs and Classical Lewis Adducts: A Domain-Based Local Pair Natural Orbital Coupled Cluster Study," *Chemistry: A European Journal*, vol. 23, no. 4, pp. 865–873, 2017, doi: 10.1002/chem.201604127.I.

[3] T. Weymuth, E. P. A. Couzijn, P. Chen, and M. Reiher, "New benchmark set of transition-metal coordination reactions for the assessment of density functionals," *J Chem Theory Comput*, vol. 10, no. 8, pp. 3092–3103, Aug. 2014, doi: 10.1021/ct500248h.
